# “Suffering as a Lifelong Companion: A Phenomenological-Hermeneutic Study of Men Living With Severe Psychiatric Illness” Lidelse som livslang følgesvenn: En fenomenologisk-hermeneutisk studie av menn som lever med alvorlig psykiatrisk sykdom

**DOI:** 10.1177/23333936211073616

**Published:** 2022-01-29

**Authors:** Anne Kasén, Terese Bondas

**Affiliations:** 1 471919 Faculty of Nursing and Health Science, Nord University, Bodø, Norway; 2 155312 Faculty of Health Studies, VID, Specialized University, Bergen, Norway; 3 56627 Faculty of Health Sciences, University of Stavanger, Norway

**Keywords:** suffering, adult men, severe psychiatric illness, phenomenological-hermeneutic, Norway, lidelse, voksne menn, alvorlig psykiatrisk lidelse, fenomenologisk-hermeneutisk, Norge

## Abstract

Accumulation of suffering in later life due to severe psychiatric illness has received surprisingly little interest in nursing research. Suffering in daily living seems to be more demanding for men, a phenomenon still debated in the literature. This phenomenological-hermeneutic study aims at describing and interpreting the perspectives of adult men and their experiences of suffering in daily living with severe psychiatric illness, diagnosed as schizophrenia. Data were collected in dialogical conversations with four men aged between 20 and 40 years, living alone in northern Norway. The themes created from the structural understanding illuminate the participants’ suffering as simultaneously struggling against the grasp of the illness and for reshaping the future. The theoretical interpretation unfolds the multidimensionality of their suffering and the need for confirmation of the suffering and reconciliation with the losses from illness, thus making reorientation to the future possible.

## Abstract (no)

Akkumulering av lidelse senere i livet på grunn av alvorlig psykiatrisk sykdom har vekket overraskende liten interesse innenfor sykepleieforskning. Lidelse i dagliglivet ser ut til å være mer krevende for menn, et fenomen som fortsatt diskuteres i litteraturen. Denne fenomenologisk-hermeneutiske studien har som hensikt å beskrive og tolke perspektivene til voksne menn og deres erfaringer med lidelse når de lever med alvorlig psykiatrisk sykdom, diagnostisert som schizofreni. Data ble samlet inn i dialogiske samtaler med fire menn i alderen 20 til 40 år, som bor alene i Nord-Norge. Temaene som skapes gjennom den strukturelle forståelsen belyser deltakernes lidelser, som å kjempe mot sykdommens grep samtidig som man strever med å omforme fremtiden. Den teoretiske tolkningen utfolder multidimensjonaliteten av deres lidelse og behovet for bekreftelse av lidelsen og soningen med tapene fra sykdom, og gjør dermed re-orientering til fremtiden mulig.

## Introduction

Suffering in daily living with a severe mental illness such as schizophrenia seems to have gained less interest among researchers than the influence by biological, psychological, and environmental causes on the outburst of the illness. In an extensive systematic review Cunningham & Peters (2014) concluded that schizophrenia is complex and seems not to be a single process with one cause but a group of diseases with similar symptoms. Differences between the sexes have also gained interest as the onset of schizophrenia seems to occur at between 21 and 25 years of age for men, with a somewhat later onset, between the ages of 25 and 30, for women (Falkenburg & Tracy, 2014; Piccinelli & Homen, 1997). Worldwide, the illness is more common among men (12 million) than among women (9 million), and approximately 1% of the population is diagnosed (WHO, 2018). Gender differences in the illness have been discussed by, among others, da Silva & Ravindran (2015), Häfner (2003), and Schön (2010), who reported poorer outcomes for men than for women. Women diagnosed with schizophrenia are also reported to have better integration in the community and a more benign course of the illness with lower levels of disability than men (Morgan et al., 2008). On the contrary, contradictory results are reported about gender differences. In a study over a period of 20 years, comparing treatment outcomes for men and women diagnosed with schizophrenia, Cechnicki et al. (2018) found no statistically significant differences between the groups. There seemed however to be a slight trend that women reported a higher subjectively assessed quality of life than the male participants in the study. The differences in social functioning in adult age between men and women in favor of women may also be explained by the later onset of the illness by women. Women may have had more time to build social networks and occupational roles, this possibly explaining the more socially dysfunctional behavior described for men (Falkenburg & Tracy, 2014; Häfner, 2003). Fleming (2004) also points out the later age of onset for the illness for women as providing them with more coping skills and life experience, all strengths that should be built on when planning for nursing interventions. In a Nordic study, Schön (2010) ponders that social and cultural expectations may also influence the recovery process, as participating in working life and providing for the family is a life ideal for many men in Scandinavia. The male participants in her study were living quite a different life from this ideal, struggling to control the symptoms of schizophrenia. The women in Schön’s (2010) study seemed less dictated by cultural expectations. In exploring occupational needs and interests in “reconfiguring maps of life” for young men living with schizophrenia, Gould et al. (2005) state that more research is needed to understand experience of illness and how to support the process of remaking life in the provision of care. Our focus in this study is on illuminating the lived experiences of suffering in the male participants’ daily life with schizophrenia from a nursing care perspective.

### Background

Severe psychiatric illness such as schizophrenia can permanently change the direction and the quality of life for the person affected and later life may be like living in another world (Mauritz & van Meijel, 2009). In their study of individuals with schizophrenia, family members, and healthcare professionals, Noiseux & Ricard (2008) described schizophrenia as a descent into hell. Life might be imprinted with doubts of self; fear of the inner, psychotic world; and an erosion of the sense of belongingness through the loss of relationships in the external world (Mauritz & van Meijel, 2009). Despite longstanding criticism of the bio-medical model, understanding of the disorder still arises primarily through (bio-)medical explanations. In turn, causation, symptoms, and treatments are increasingly sophisticated and well-known, while understanding of other aspects of the disorder, especially the intersubjective experience of people living with schizophrenia, remains fragmented (Geanellos, 2005).

“Illness engulfment,” a concept formed to describe the damage to an individual’s concept of self caused by schizophrenia, has been described as a process with protective and destructive potential. Schizophrenia has a significant risk of damaging an individual’s self-concept (Vining & Robinson, 2016). Harrison & Gill (2010) described stigmatization as experiences of devaluation in work life, social exclusion, and disempowerment. The destructive aspects often seem to dominate, with massive losses in identity, difficulties in relationships, and a change of the primary self-concept into a post-diagnosis identity. Kaite et al. (2015) describe experiences of being an outcast. Experiencing cognitive disparities due to mental illness and imagining oneself as “odd” may increase suffering through social and existential alienation (Erdner et al., 2005). Olçun & Şahin Altun (2017) found a significant negative correlation between internalized stigma and hope, and a significant positive correlation between the stigma resistance of patients and their hope levels. Harrison & Gill (2010) also found a close connection between the diagnosis, negative attitudes in society, and feelings of pessimism among healthcare professionals, leading the individual to conceal the illness by withdrawing from relationships. The loss of important relationships can be twofold: the loss of intimate relationships with friends and loved ones and the loss of relationships within the community and in support groups (Baker & Procter, 2015). Similarly, Avieli et al. (2016) described an “all-consuming,” multifaceted suffering invading all aspects of life and influencing, especially, working life, intimate relationships, and housing. Walton (2000) further described the experience of schizophrenia as living with the prejudice of others, being fearful of others, feeling uncomfortable in the company of others, trying to stay engaged with others in the world, depending on others for help, and trying to find others who understand.

### Accumulated Suffering in Living With Schizophrenia

As an individual with schizophrenia ages, suffering accumulates through years of symptoms, possible hospitalizations, and side effects of medication. Avieli et al. (2016) recommended that suffering and endurance should be acknowledged and reaffirmed in the narrative of life during all therapeutic work. Recent studies (Avieli et al., 2016; Moonen et al., 2016) pointed out that interpretations of a person’s suffering could likely provide deeper insights into the meaning of the lived experience. Supporting this assumption, Ko et al. (2014) found that reflections on the suffering and of the spiritual beliefs in the patient’s own cultural context could work as a means of finding ways of reintegration through regaining control and finding inner peace. In a meta-ethnography of 17 qualitative studies, Kaite et al. (2015) presented the profound loss of identity, pain of having had one’s life stolen, and an ongoing struggle for reconciliation with the self and the illness as core themes of living with severe mental illness. Suffering as a phenomenon itself has been shown to have negative, progressive consequences, such as pain, loneliness, alienation, and poor self-esteem (Al Kalaldeh et al., 2018). Kaite et al. (2015) asserted that the diverse experiences of patients with severe mental illness have not been considered in treatment and care. The personal experience of having schizophrenia has gained sparse interest in research (Walsh et al., 2016). Even less attention seems to have been directed toward exploration of an individual’s suffering from severe psychiatric illness, especially the meaning from the male perspective. Therefore, further research on suffering, and demands of living with the illness, is needed.

### Theoretical Perspective

The theoretical perspective chosen to deepen the understanding of male individuals’ suffering from living with schizophrenia emanates from psychiatric nursing and caring theories. Florence Nightingale suggested a perspective of illness as suffering, irrespective of its physical or mental nature, in her notes published as early as 1860. Nightingale (1969) observed that the patient’s suffering may not entirely be caused by the disease itself but by something else, often a lack of nursing care. Among later nursing theorists who saw suffering as a central concept, there seems to be a slightly different perspective on the reasons for suffering. Travelbee (1971) pointed out the personal loss of something significant as a reason for suffering to occur. She stated that when we as human beings care for something, there is always the possibility of experiencing suffering when we lose or are in danger of losing the thing cared for. Eriksson (2006) viewed all human beings as potentially suffering beings: suffering is part of life and may actualize at times when illness or losses occur. The drama of suffering is, according to Eriksson (2006), the person’s struggle for recognition and confirmation of his/her suffering-a cry for another person to be a co-player, to see and to hear the suffering. Through this drama, the suffering can be alleviated, and the person may reach reconciliation.

### Aim of the Study

The aim of this study is to describe and interpret the perspectives of young adult men and their experiences of suffering in daily living with severe psychiatric illness, diagnosed as schizophrenia. The research question was: What are young adult men’s experiences of suffering when living with severe psychiatric illness?

## Methods

We chose a hermeneutic-phenomenological approach to understand suffering (Kvale & Brinkmann, 2014). This approach was recommended by Al Kalaldeh et al. (2018) and Graor & Knapik (2013) to give voice to persons living with schizophrenia in order to affect practice. However, the approach is not without challenges, especially ethically, calling for enhanced informed consent. The authors further suggest an effort to balance researcher–participant inequality by stating the researchers’ dependency on what the participants want to tell. Narrative data collection, suggested by Kirkpatrick (2008) can provide access to the human experience of severe mental illness through listening and understanding, and may add to a recovery-oriented paradigm, instead of one focusing on pathology and chronic illness. Inspired by these thoughts on data collection among vulnerable participants, we undertook dialogical conversations with the participants. Dialogical conversations are also in accordance with the overall phenomenological-hermeneutic approach chosen for the potential to describe and interpret lived experiences of suffering.

### The Participants

As our research focus was young adult, male participants living with severe psychiatric illness, such as schizophrenia, our inclusion criteria were: Male persons of age 20–40 years, living in their own home and with the diagnosis schizophrenia, willing to participate, and in a stable phase of the illness that made informed consent and participation possible. The age inclusion criteria were important to capture the participants’ thoughts and dreams about the future. Likewise, was it important that the participants were living at their own, not as patients at an institution in order to capture their ordinary daily living. Exclusion criteria were symptoms from illness that made informed consent and participation impossible. We searched for participants by first contacting head managers at the mental health municipal healthcare service in different municipalities in Norway. The contact was taken both in e-mail, and a few days after by phone. The head managers then contacted psychiatric nurses in their area and asked for possible participants. This nurse was the person asking the participant and giving him information about the study. Some head managers thought that men would be reluctant to share their experiences with a researcher who was likely to be viewed as a stranger. This showed to be partly right, as there was some hesitation among asked persons meeting the inclusion criteria to participate in the study, but as soon as agreement on participation was reached, and the participants met with the researcher a rich dialog unfolded and no one withdraw from the study.

The participants, four men aged between 20 and 40 years were all living alone in their own homes in small towns in northern Norway. The participants were unknown to each other, and they were also unknown to the researchers. Three of them had their parents and one also had siblings living in the neighborhood, while one participant did not have relatives in the same town. The participants had not been married or had children, and none was in an intimate relationship. They described their friendships as limited and their contacts as sparse. One participant described his voluntary assistant as a friend rather than a helper. The participants had been diagnosed with schizophrenia between the ages of 15 and 25, and all of them had had several previous acute episodes of the illness demanding hospital care. All participants had finished elementary school. Two participants had completed three years of high school education. Two participants had had their education interrupted due to their illness. One participant had to interrupt his university education; the illness had stopped him. At the time of the interviews, two of the participants were receiving disability pensions and one participant was in an active rehabilitation process. Only one of the participants was participating in ordinary work life.

After agreement with the participants, one of the researchers met them individually in their own homes. She had extensive experience as a psychiatric nurse caring for adults suffering from severe mental illness. She gave them time and created rapport in a trustful situation before conducting dialogical conversations (Graor & Knapik, 2013; Kirkpatrick, 2008). The conversations focused on living with severe psychiatric illness and the influence of suffering from the illness and its impact on relationships and interaction with other persons. The conversations were open, and the researcher tried to follow the participant’s descriptions of his life by adding questions as necessary. The conversations lasted from 1.5 to 2 hours and generated rich material.

We adopted the concept of “information power” by Malterud et al. (2016) in determining the sufficient number of participants. Information power in our study is supported by the theoretical framework of suffering, the rapport created by the researcher during the dialogs with the participants, conducted in their own homes which elicited long in-depth narratives of suffering affecting daily life. Information power is further strengthened by the use of a phenomenological–hermeneutic analysis strategy to illuminate nuances of young adult men’s suffering in everyday life when struggling with prolonged severe psychiatric illness, such as schizophrenia.

### Data Analysis

In the phenomenological-hermeneutic method (Kvale & Brinkmann, 2014) chosen for this study, there are three levels of description and interpretation. At the first level (self-understanding), voice is given to the participants and their understanding of their suffering. The narrative, *Geir’s story* is constructed by the authors after the first readings, condensing all the participants’ experiences. Geir is a common male name in Norwegian, thus selected in order to secure anonymity for the participants. The second level (structural understanding) begins with several readings of the transcribed manuscripts. Then sub-themes and themes are constructed and named, and finally they are interpreted as an overarching theme which embraces them. At the third level, the structural understanding is enriched through theoretical interpretation using Travelbee’s (1971) and Eriksson’s (2006) theories on suffering.

### Ethical Considerations

As the participants all lived with a severe mental illness, schizophrenia, the invitation to participate in the study was carefully planned. At the time of the interviews, all participants were able to make the decision to participate and provide informed consent during the conversations. None withdrew from the study during or after the data were collected. The Norwegian Centre for Research Data (NSD) and the Regional Committee for Medical and Health Research Ethics (REK) approved the study. The participants were informed both in writing and orally, and they all signed informed consent forms. The researcher was also prepared so that she could contact the participant’s own psychiatric nurse if he showed any sign of stress or additional suffering related to the dialogical conversation. There was no need to do this. Due to ethical considerations, all sensitive data were removed, and all living conditions are described in a general manner.

## Findings

The findings are reported according to the phenomenological-hermeneutical method described earlier. The interpretation starts from the description of the participants’ own understanding and proceeds through a structural understanding to a theoretical interpretation.

### Level 1: The Participants’ Life Environments and Their Self-Understanding

Three participants lived in small cities, and one in a rural environment. All participants had their families living nearby, none were in an intimate relationship, and only a few friendships were described. Two participants received disability pensions due to their illness, one was in a rehabilitation process to be able to live more independently, and one was working. All had contact with the local psychiatric healthcare institution for care and medication. All also received help with housekeeping from the community. All participants had creative interests. Two played music and composed, and two worked on carpentry as a hobby. Both hoped one day to open a joinery of their own. Outdoor activities such as hiking in the mountains were also mentioned. Christian faith was an active part of life for two of the participants.

### Geir’s Story

The description of Geir’s understanding (level 1) is constructed based on all the participants’ stories.

I was hit by schizophrenia in my teens. It was like a breakdown. I couldn’t pass any exams from high school because of the illness and my dream of a career in the army was also shattered. After that, I have been in a psychiatric hospital several times. These were fearful experiences with a lot of medicine and side effects from them. It is horrible! For a long time, I tried to avoid doctors as they always prescribed more medicine for me.

I don’t like crowds; they fill me with agony. Agony is the worst with my illness; it hinders me from doing things, like going to the movies. I hear voices almost all the time, but they are more present when I am alone. The illness has distorted my brain and my whole life. I long for someone to love, but I suppose no one will ever love someone as distorted as me. I try to manage my agony with the medicines my doctor prescribes to me. I meet him twice a year. Springtime is the best time of the year for me; life is somehow easier then.

### Level 2: The Structural Understanding

**Figure 1.**
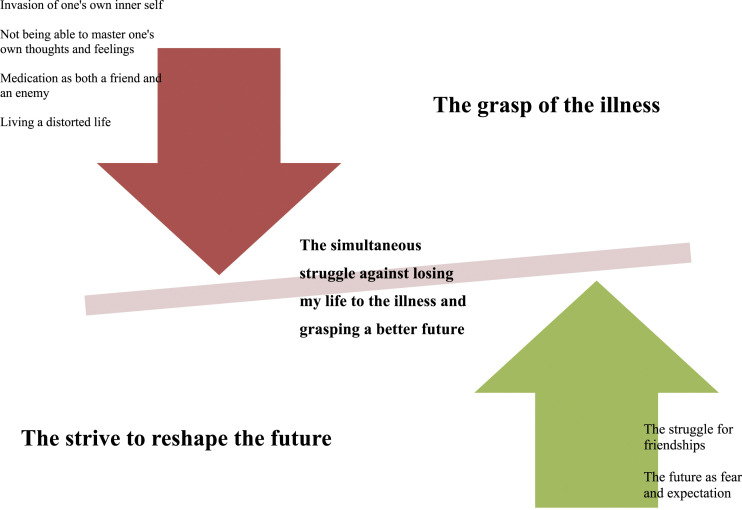
Themes, sub-themes and the overarching theme in the structural understanding.

The overarching theme created from the structural understanding illuminates the participants’ suffering in their struggle against the illness’ grasp of life, and at the same time, a forceful struggle to reshape the future. Figure 1.

This struggle can also be interpreted as seeking harmony, balancing between the forces from the illness taking command of life and striving for a future with the illness, but with family and friends. The participants’ balancing is active, at the same time resisting the power of the illness invading every dimension of their life and striving for a satisfying life, wishing, and hoping to be like everyone else.

## The Grasp of the Illness

The illness can be described as a destructive power grasping the participants’ lives, here illuminated by four sub-themes: invasion of one’s own inner self, not being able to master one’s own thoughts and feelings, medication as a friend and an enemy, and living a distorted life.

### Invasion of One’s Own Inner Self

All four participants described terrifying memories of hearing voices, seeing figures “from another world” or of smell and taste being distorted, sensations that they could not control or stop. These experiences were like an invasion of one’s own inner self. They all described these sensations as scary, contributing to constant feelings of fear and agony. Hearing voices stemming from “inside” disturbed concentration and made conversation with other people hard, as the participants listened to “several voices and have to decide which to answer.” The intensity of these sensations varied from modest disturbance of daily living to massive agony and an overwhelming fear of death:

I felt that my body was invaded in some way and my body changed form. I was lying on a sofa and spiders were crawling all over me and into my body … I was screaming in fear and then my body became like an open grave and I fell into it.

Participants felt as if their bodies were being invaded and destroyed without any defenses to stop this invasion or restore their bodies. Another participant concluded, “It is like being in a horror movie while being awake.”

### Not Being Able to Master One’s Own Thoughts and Feelings

All the participants described difficulty gaining control of their own thoughts, a constant fight to think clearly and to get their thoughts in the “right direction.” They struggled to concentrate and were afraid that others could manipulate their thoughts or be able to read their thoughts. Feeling that others can read one’s thoughts increases feelings of being skinless and transparent to others’ opinions and influence. One participant stated:

I have been in the hospital for two-and-a-half years due to psychotic symptoms. I am suffering from schizophrenia. You can be awfully afraid, have distorted thoughts, and things can get so confused as I feel that others can read me. I am working with myself all the time, as others also probably do. Of course, I want to keep my dignity.

Not being able to control one’s own thinking also interferes with the individual’s relationships and affects the feeling of dignity, of not being able to connect with others.

Ordinary feelings can also change in form and intensity due to the illness and be felt as strange and overwhelming. This can be fascinating and scary, as the world we used to know is no longer the same. One participant stated, “My feelings were changing constantly, and I had sensations of many feelings at the same time, like a synthesis or so.”

### Medication as a Friend and an Enemy

The different kinds of medications used in psychiatric care to reduce symptoms can help in mastering daily life, but also cause a loss of feelings and flexibility, and make thinking more mechanistic with a loss of nuance and color. One participant described how the medication helped him maintain normal sleeping habits, and he stated, “If more than a week without medicine, my concentration begins to fail, and the bad thoughts are back.” For another participant medication was more of an enemy, making him more tired, “the muscles slacker, and life grayer.” Being tired can contribute to passivity and an inability to connect with others, and therefore, may not only be a sign of the illness but also a side effect of the medication.

### Living a Distorted Life

The illness imprinted the four participants’ lives in different ways. They described interrupted education and work careers ended, like “dreams washed out in the sink”. One participant described how the illness has destroyed his brain and distorted his whole life, as he goes on living like the living dead:

There is something wrong in my brain; it is destroyed. I think a lot about how my life could have been without this illness, without schizophrenia, what it would have been like. I had planned to work in the military, but it was not possible after the illness struck.

The lives of the participants seemed to be on pause, and they talked about the fear of never being able to live like a normal person again, the fear of not getting a wife and being able to establish a family of their own. They felt lonely and longed for relationships with others but avoided contact with others in fear of being seen as odd.

For one of the participants, the spiritual dimension in life was also distorted, as he felt that God had deserted him, and his illness was punishment for his previous sins. The plague for him was twofold, as he did not know what he had done to be punished in this way.

## The Strive to Reshape the Future

The structural interpretation also revealed the participants’ striving to reshape their future. The struggle for friendships and how the future entailed both fear and expectation are two sub-themes that illustrate this interpretation.

### The Struggle for Friendships

All participants described how they longed for friends and how they struggled to form friendships. None mentioned loneliness or being alone, but all discussed isolation and the fear of closeness in relationships with other persons. One described how, despite the agony, he sought the company of other people:

The agony is worst, I don’t dare to be out in the town, but I force myself, you must do something yourself. When I get out in the streets of my hometown I am agonized, there are so many people I get nervous when there is a lot of others.

The participant tried to overcome the agony and perhaps win over the isolation at home. Another participant also pointed to the lack of abilities needed in relationships with others because of the long time isolated in the illness’s grasp:

I am inhibited in social relations; I have been alone so much that I easily “get on a wrong trace”. I have never learned to defend myself, either, or if in a dispute, I can’t do anything but lie. I do not have anything worth defending. I am a loser.

### The Future as Fear and Expectation

The participants often reflected about the future: “What will it bring? Maybe I can get better, maybe have a family?” One participant contended that hope is a strength within all of us, hope for a better future. Hope can make us endure almost anything. Another participant described how he had got help in mastering his difficulties and thus was able to manage his life, to have dreams and visions for the future. He hoped for the possibility of living an ordinary life in which other people respect his illness as he respects them. Another participant feared the possibilities of the future. What if other people still see him as odd and not as who he really is?

### Level 3: The Theoretical Interpretation

Our understanding of the overarching theme from the structural analysis as the participants’ suffering as simultaneously struggling against the grasp of the illness and reshaping the future can be further illuminated in Travelbee’s (1971) theory. The participants’ descriptions of significant losses in their lives as education, work, and someone to love to the illness, can all contribute to suffering. Interpreted from Eriksson’s (2006) theory the participants’ struggle can be seen as a movement toward health as endurable suffering. In a sense, this means reconciliation with the circumstances, to live with the illness, but not acceptance.

At first glance the theme “the grasp of the illness” as described in the structural interpretation, seems to be “suffering related to illness” in Eriksson’s (2006) theory. The suffering from the symptoms of the illness is captured in sub-themes as “not being able to master one’s own thoughts and feelings” and shortcomings caused by the illness itself when “living a distorted life” or exemplified by the description of an “invasion of one’s own inner self.” But at closer inspection, the theme also captures existential suffering, suffering stemming from knowledge of life and death (Eriksson, 2006). The sub-theme “invasion of one’s own inner self” also holds a strong fear of death. Eriksson’s concept “existential suffering”, that is, suffering related to the conditions of life itself, also shines through in the sub-theme “living a distorted life.” The participants longed for communion, to belong in society, in working life, and, especially, in close relationships.

Losses such as a profound reorientation of visions for later life, as described in the sub-theme “future as fear” in the theme “the struggle for the future,” can also be interpreted as existential suffering (Eriksson, 2006). For reorientation to be possible, the person needs to mourn the loss and to get confirmation that mourning the loss is allowed (ibid.). Only then can a new orientation take form. For the participants in our study to endure the different aspects of suffering imbedded in their lives, they need confirmation that their suffering is real, they need to “play the drama of their suffering”, not only for an audience listening but with a confirming counterplayer, to speak from within Eriksson’s (2006) framework. Who can then be the counterplayer in their suffering? Is this a call for their families or the sparse friends nearby? Interpreted against our theoretical framework, this is the mission of healthcare personnel, especially psychiatric nurses who can be the counterplayers in the suffering person’s drama.

Travelbee’s (1971) theory can also add to our understanding of the importance for bringing hope into the suffering person’s life by seeing each person as a fellow human being. The nurse’s invitation to the lonely person to fellowship in a caring relationship, in which they together can examine the different and often frightening experiences of the illness, may increase the person’s ability to manage such experiences. The drama of suffering as described above by Eriksson (2006) can take place in this fellowship, and make room for reconciliation, actualization of self and reshaping of the future.

But as Nightingale (1969) already pointed out, also lack of nursing care can cause suffering. Striking in the participants’ narratives is the absence of healthcare personnel generally and particularly psychiatric nurses. Or if they are present, they do not appear to be central in the participants’ lives. Absence of a caring relationship where suffering can be alleviated can in Erikssons’ theory (2006) be understood as suffering related to care.

In conclusion the theoretical interpretation unveils the suffering in the participants’ struggle against the illness and towards a reshaping of the future as multidimensional: suffering from the illness itself and its symptoms, but also suffering the losses in life itself the illness has caused. Their suffering is also existential, evoking questions of life and death. Additionally, it seems like their suffering is made worse by an absence of caring relationships with healthcare personnel where they could get confirmation of the suffering and mourn the losses the illness has brought, thus making reconciliation and reorientation to the future possible.

## Discussion

In this study we describe and interpret the perspectives of young adult men and their experiences of suffering in daily living with severe psychiatric illness, diagnosed as schizophrenia. Our main finding, the participants’ suffering as simultaneously struggling against the grasp of the illness and for reshaping the future, illuminates the multifaceted nature of their suffering and the importance of the participants reaching a new equilibrium, which according to Eriksson’s theory (2006) can be interpreted as endurable suffering and thereby as health.

### Suffering From Illness and the Losses it has Brought

There had been several losses in the lives of our participants because of the illness, such as loss of education, lack of work and careers that had been hoped for, lack of friends, and lack of intimate relationships. Losses that are interpreted as adding to suffering from their illness. They longed to be like other people, to have friends, family, and a meaningful work. But at the same time, they describe the struggle to connect with others. The suffering associated with these losses may be particularly significant for men. The men in this study were between 20 and 40 years of age when there are social expectations that these be productive years for men, with important life tasks; a sharp contrast to the restricted and socially isolated lives they appeared to be living.

The struggle for friendship described in the present study can be found in what Avieli et al. (2016), Baker & Procter (2015), and Mauritz & Van Meijel (2009) described as significant feelings of loss: loss of social life, and loss of hope to be a partner and a parent, the loss of both types of intimate relationships. Relationships are seen as one of the most crucial yet challenging elements of recovery and well-being for people affected by mental illness, findings that are fully in line with the present study.

The struggle for friendships can however be hindered by fear of being seen odd or rare as described by our participants, and as a result they may avoid contact. Vining & Robinson (2016) identified a phenomenon named “illness engulfment”, a total reorganization of an individual’s self-concept around the experience of living with schizophrenia. Erdner et al. (2005) also found a contradiction between the person’s longing for relationships with others while lacking a genuine interest in the other person because of an overwhelming need to be accepted by the other.

Buckland et al. (2013) and Walton (2000) contended that the prejudice of other persons regarding schizophrenia can add to fear and discomfort, hindering a sense of belongingness. The impact of the stigma was further explored by Harrison & Gill (2010), who concluded that it is important to overcome it with the help of nurses.

### Suffering Related to Care

The participants in the present study also describe a struggle to reshape their future. Expressions of grief seem important for coming to terms with the illness and finding a new equilibrium, and hope seems to be very important for the recovery process, as the theoretical interpretation from Travelbee’s (1971) theory indicates. In Noiseux & Ricard’s (2008) study prerequisites for recovery from schizophrenia is an igniting of a spark of hope that can activate the instinct to fight back and discover keys to well-being. The importance of hope for recovery of schizophrenia is also confirmed by among others (Barut et al., 2016),van Gestel-Timmermans et al. (2010) and Kylmä et al. (2006). Noh et al. (2008) underlines that hope for people living with schizophrenia is similar to hope for all human beings, it originates from interpersonal relationships, from loving and being loved.

The present findings support Honey et al.’s (2015) and Kaite et al.’s (2015) work on the importance of supporting recovery through meaningful relationships, activities, and support of the development of a worthy sense of self, so that individuals can manage their everyday lives. The participants in Mauritz & Van Meijel’s (2009) study dealt with their losses by accepting their diagnosis and treatment, identifying with other patients, learning about schizophrenia, and searching for meaning.

However, the personal caring relationship with a nurse where hope can be nurtured and the existential suffering can be processed, as described by Moonen et al. (2016) seems rather absent in our participants’ lives. This absence is in our theoretical interpretation described as a suffering related to care (Eriksson, 2006) One may wonder if a pessimistic view on the outcome of schizophrenia, a view described in Ross & Goldner’s (2009) review of studies relating to negative attitudes and discrimination of psychiatric illness within the nursing profession, may have contributed to this. The participants in our study have struggled with the illness for between 10 and 15 years, but they were still dreaming of a better, “ordinary” future. The healthcare system may however have lost hope for their recovery.

### Reconciliation With Suffering From the Illness

Reconciliation with the suffering from schizophrenia as a part of life, rather than as something defining the person, is indicated in our theoretical interpretation (Eriksson, 2006) to overcome the struggle of living with the illness. Walsh et al. (2016) also emphasized acceptance of the diagnosis with schizophrenia as a part of life, rather than as something defining the person to overcome the struggle in living with the illness.

In the present findings, a paradoxical longing for relations while expecting rejection by others appeared; the future seems to be both fearful and filled with expectations. Lifelong suffering from severe psychiatric illness may foster social inhibition. The same kind of suffering is visible in a meta-synthesis (Kaite et al., 2015) describing the ongoing struggle for reconciliation with the self and the illness, alongside with loss of identity, pain of having had one’s life stolen, and experiences of being an outcast. Training in social skills can be considered one path in psychiatric nursing toward mastery of life in the community for patients living with chronic schizophrenia (Seo et al., 2007). Bengtsson-Tops (2004) added community-based nursing services as effective for mastery of psychiatric symptoms, social skills performance, and needs for care and support in living, nutrition, and daytime activities. Seo et al. (2007) found that social skills training as a nursing intervention may improve social skills and self-esteem. Medication, self-management, and self-administration can be supported by teaching, supporting, and partnering with patients (Hochberger & Lingham, 2017). Gould et al. (2005) suggest that occupational therapy also can provide tools in his reconfiguration of the map of life and a transition back to working life and society. Mauritz & Van Meijel (2009) concluded that, in addition to optimal assessment and treatment of symptoms, information about symptoms, treatment and its effects, and prognosis, individuals need opportunities to identify with other patients, strengthening of social support, and a relationship of trust with care providers based on an accepting attitude. To achieve a life in reconciliation with the illness, the person living with schizophrenia need possibilities to reinterpret his/her life in a new way in a caring relationship with a nurse.

### Limitations

The context for our study, a Nordic welfare country with free healthcare services, could be a limitation for transferability of the findings. The participants were all men; however, neither sex nor gender-related influences were a primary focus (Fausto-Sterling, 2012), and more research is needed to explore the influence of these factors on suffering in the context of schizophrenia.

The small number of participants in the study could be considered a limitation, but the rich data emanating from one conversation with each participant is a result of the rapport created by the researcher and made room for narratives of living with suffering to unfold. There is, however, a need for further studies of immigrant men and their experiences of living with suffering related to severe psychiatric illness, as well as follow-up studies. Trustworthiness was addressed by dialogs in the research team where two had experiences as psychiatric nurses and one of us had been a nurse leader in psychiatric care. We had extensive experience of qualitative research and first worked one and one with the phenomenological-hermeneutical analysis, then two of us discussed the interpretations using analytical notes. The third person then reread the analysis to add to/refine the result.

## Conclusion

The suffering of young adult men living with schizophrenia is interpreted in the light of Eriksson’s (2006) and Travelbee’s (1971) theories of suffering. Here the multidimensionality of suffering in living with severe psychiatric illness is unfolded. The fellowship in a caring relationship with a nurse can be a frame for learning to master the illness and alleviate suffering toward reconciliation. The reshaping of life in reconciliation with the illness may take place. Living a fuller life, while still suffering from the illness, as a lifelong companion, can be a possible future.
